# Anti-KLHL11 antibody encephalitis without detectable malignancy: a case report and literature review

**DOI:** 10.3389/fimmu.2026.1913121

**Published:** 2026-09-17

**Authors:** Chenxin Yao, Mengtan Cai, Zixuan Shen, Xiaoxu Zhang, Jie Cao

**Affiliations:** Department of Neurology, the First Hospital of Jilin University, Jilin University, Jilin, Changchun, China

**Keywords:** cerebellar ataxia, epileptic seizures, immunotherapy, KLHL11 antibody, occult malignancy

## Abstract

**Background:**

Anti-Kelch-like protein 11 (KLHL11) antibody encephalitis, first reported in 2019, is a rare autoimmune disorder typically associated with seminoma. This disorder exhibits a striking male predominance, with cerebellar ataxia as the hallmark manifestation, whereas seizures and altered consciousness occur less frequently. Cases without detectable malignancy account for fewer than 20% of reports, and their clinical manifestations, treatment responses, and outcomes have not been systematically characterized.

**Methods:**

We report a case of a 62-year-old male with anti-KLHL11 encephalitis and no detectable malignancy, who initially presented with recurrent generalized tonic-clonic seizures (GTCS), followed by intermittent seizures and progressive neurological deterioration. A systematic search of published cases with narrative synthesis was conducted to analyze the clinical presentation, neuroimaging features, treatment responses, and outcomes of similar reported cases.

**Results:**

We analyzed 13 cases of anti-KLHL11 encephalitis without detectable malignancy. The most common clinical manifestations were ataxia (9/13), dysarthria (8/13), and gaze palsy/nystagmus (6/13). Intracranial pressure (ICP) (median: 165 mmH_2_O, range: 60–200 mmH_2_O), cerebrospinal fluid (CSF) leukocyte count (median: 9 × 10^6^/L, range: 1–46 × 10^6^/L), and CSF protein level (median: 0.50 g/L, range: 0.27–1.32 g/L) were also measured. The median serum anti-KLHL11 antibody titer was 1:100 (range: 1:30–1:160,000), while CSF anti-KLHL11 antibody titer was 1:10 (range: 1:1–1:32). Electroencephalography (EEG) was normal in 2 cases, showed epileptiform discharges in 2, diffuse background slowing in 2, and sleep-related central hypoventilation in 1. Brain magnetic resonance imaging (MRI) was unremarkable in 3 cases. Seven cases (53.8%) presented with T2/fluid-attenuated inversion recovery (FLAIR) hyperintensities, 4 cases (30.8%) showed cerebellar/brainstem atrophy, 1 case (7.7%) presented with acute cerebral infarction. Following immunotherapy, 6 patients achieved clinical improvement, 3 patients remained clinically stable, 4 deteriorated clinically, of whom 2 died of central respiratory failure.

**Conclusion:**

Anti-KLHL11 encephalitis without detectable malignancy cannot be reliably distinguished from tumor-positive cases based solely on clinical symptoms and neuroimaging findings. The gold standard for differential diagnosis remains comprehensive tumor screening and long-term follow-up. Extensive white matter and deep gray matter involvement, together with prominent seizures, may be more prevalent in patients without detectable malignancy, although these observations require validation in larger comparative cohorts.

## 1 Introduction

Kelch-like protein 11 (KLHL11) is a component of the E3 ubiquitin ligase complex (1) and is highly expressed in the testis and brain (2). Mandel-Brehm et al. first described anti-KLHL11 antibody encephalitis in 2019 (3), identifying it as a paraneoplastic autoimmune disorder linked to testicular seminoma with primarily T-cell-mediated neuroimmune pathogenesis (4). Because of its extremely low incidence and nonspecific clinical presentation—typically ataxia and vertigo—KLHL11 encephalitis is frequently misdiagnosed as ischemic stroke, multiple sclerosis, or amyotrophic lateral sclerosis (5), which often results in delayed initiation of appropriate treatment. Studies demonstrated that 16–19% of cases have no detectable underlying malignancy (1, 6). Although cases without detectable malignancy have been reported previously, their clinical manifestations, neuroimaging features, treatment responses, and outcomes have not been comprehensively summarized. We report a case of anti-KLHL11 encephalitis in a patient without detectable malignancy who presented with recurrent generalized tonic-clonic seizures (GTCS) as the initial manifestation. We also conducted a search of the PubMed and Web of Science databases, aiming to summarize the clinical manifestations, neuroimaging findings, treatment responses, and outcomes of this malignancy-negative phenotype of the disease.

## 2 Materials and methods

In accordance with the Preferred Reporting Items for Systematic Reviews and Meta-Analyses (PRISMA) guidelines, we conducted a comprehensive literature search in the PubMed and Web of Science databases from January 1, 2019, to May 1, 2026, using the search terms “KLHL11” or “Kelch-like protein 11”, to identify cases of anti-KLHL11 encephalitis without detectable malignancy. The literature screening process is shown in **Figure 1**. Inclusion criteria: (1) Anti-KLHL11 IgG positivity in serum and/or cerebrospinal fluid (CSF); (2) No evidence of detectable malignancy identified on at least one advanced imaging modality (positron emission tomography-computed tomography [PET-CT] or contrast-enhanced computed tomography [CT]) and/or testicular ultrasound; (3) Age ≥ 18 years; (4) Complete information available on clinical manifestations, imaging findings, treatment, and prognosis. Reports lacking detailed clinical information, radiological findings, or laboratory data were excluded. Duplicate reports and conference abstracts were also excluded. Our institutional case was included as case 1 in this study. The previously reported cases were designated as case 2 (6), case 3 (7), case 4 (8), case 5 (9), case 6 and 7 (10), case 8 (11), case 9 (12), case 10 and 11 (13), case 12 (14), case 13 (15). A summary of all enrolled cases is presented in **Table 1**. This study has received ethical approval from the Ethics Committee of the First Hospital of Jilin University (Approval No.: 2026-337).

**Figure 1:**
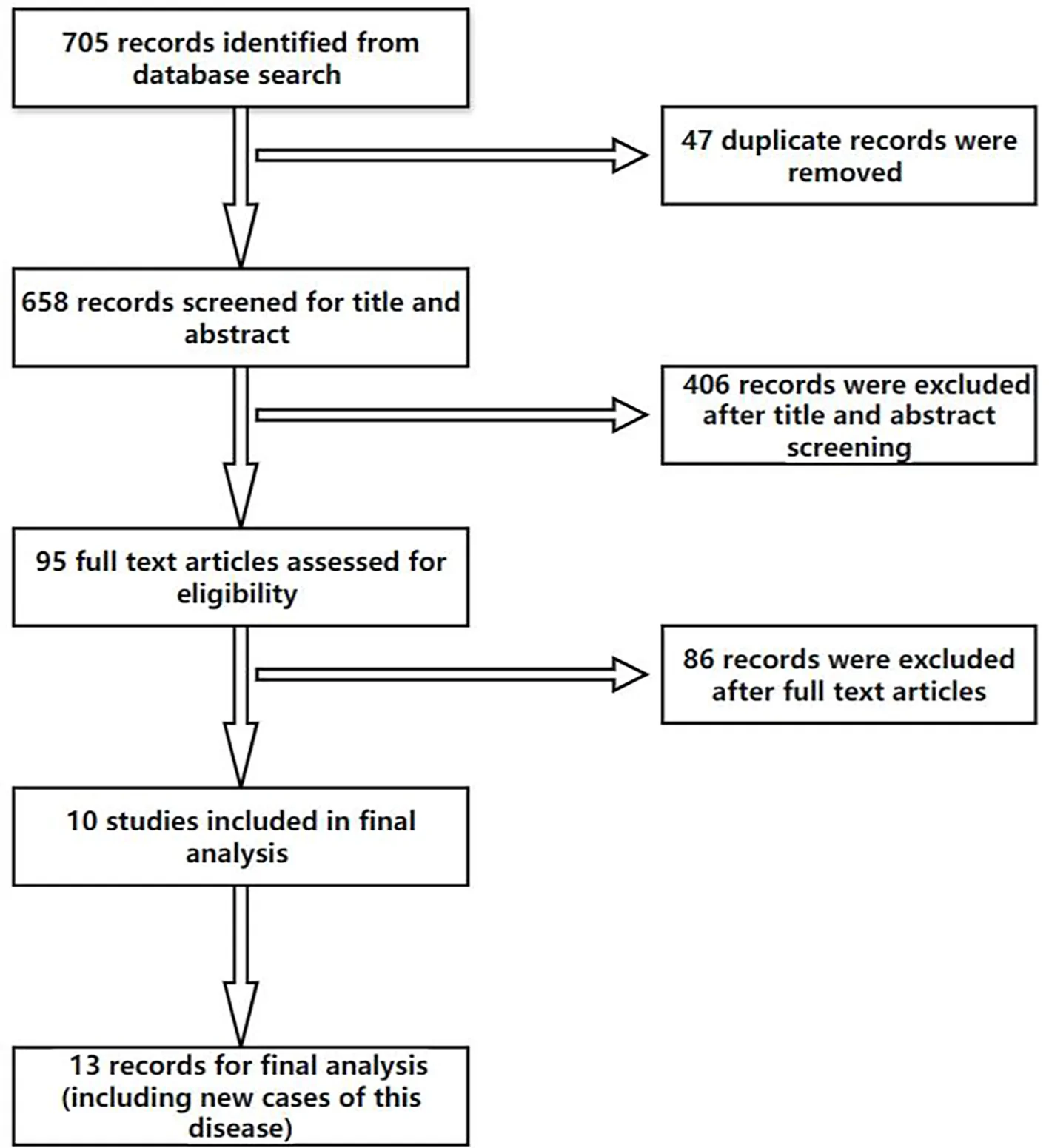
PRISMA flow diagram for study selection.

**Table 1:** Summary of cases included in this study.

NO.	Age	Sex	Clinical symptoms									CSF			KLHL11		MRI	EEG	Treatment	Follow-up
			Ataxia	Dysarthria	Gaze/nystagmus	Diplopia	Epilepsy	Vertigo	Hypoacusis	Cognitive impairment	Other symptoms	ICP	WBC	Protein	CSF	Serum				
1	62	M		+	+		GTCS				Altered consciousness	200	1	0.27	1:30	1:100	Acute left periventricular and corona radiata infarction	Bilateral frontal epileptiform discharges	IVIG	2 months
2	43	M	+	+	+	+							11	0.5	–	1:160000	Cerebellar atrophy	NR	IVMP,PSL,RTM,AZA,CTX	28 months
3	50	M	+	+		+		+			Limb weakness, hypogeusia				+	+	Punctate brainstem and cervical cord lesions (salt-and-pepper)	Normal	PDN	9 months
4	33	M	+	+					+		Sexual dysfunction		8	0.35	–	+	Bilateral hippocampal/amygdala T2 hyperintensity	Normal	GC,IVIG,PE,RTM,AZA,CTX	NR
5	66	F	+				FAS				Narcolepsy, sleep disorders, hallucinations	165	1	0.28	1:1	1:100	Normal	Epileptic waves in the bilateral temporal and sphenoid regions	IVMP,PDN,IVIG,OFA	21 months
6	74	M					+	+		+	Limb weakness		1	0.55	–	1:30	Multifocal T2/FLAIR hyperintense foci	NR	GC, IVIG, PE, EFG	2 weeks
7	50	M	+	+					+	+	Hyposmia	N	1	N	1:1	1:100	Cerebellopontine atrophy with T2/FLAIR hyperintensities	Sleep disturbances; moderate sleep-related central hypoventilation	Refuse	3 months
8	70	M			+		FAS				Unresponsiveness, headache, anterograde amnesia,behavioral abnormality	N	N	High	1:10	1:320	Left frontoparietal T2 hyperintensity	Diffuse background slowing	IVMP, PDN,IVIG, AZA,EFG	26 months
9	65	F								+	Narcolepsy	60	9	0.7	1:32	1:320	Left frontal subcortical patchy T2/FLAIR hyperintensity	Widespread continuous delta-theta activity, low-medium amplitude	IVMP	13 months
10	31	M	+	+	+	+		+	+		Limb weakness, spasticity		46	1.32	+	+	Vermian and middle cerebellar peduncle FLAIR hyperintensities with progressive cerebellar and brainstem atrophy	NR	IVIG,IVMP,PE	2 months
11	65	M	+	+						+			16	0.4	+	+	Normal	NR	IVMP,PE	NR
12	58	M	+	+	+	+					Limb spasms, sexual dysfunction, dysphagia				–	+	Diffuse cerebellar and brainstem atrophy (midbrain and medullary predominance) with periventricular and deep white matter lesions	NR	IVIG,PE,OCR,IBR	NR
13	37	F	+		+			+			Limb tremors		33	0.57	+	+	Normal	NR	IVMP,RTM	4 years

M, male; F, female; GTCS, generalized tonic–clonic seizure; FAS, focal aware motor onset seizure; ICP, intracranial pressure; IVIG, intravenous human immunoglobulin; PDN, prednisone; GC, glucocorticoids; PE, plasma exchange; RTM, rituximab; AZA, azathioprine; CTX, cyclophosphamide; IVMP, intravenous methylprednisolone; OFA, ofatumumab; EFG, efgartigimod; PSL, prednisolone; OCR, ocrelizumab; IBR, ibrutinib;NR, not reported; case2 (6); case3 (7); case4 (8); case5 (9); case6 and 7 (10); case8 (11); case9 (12); case10 and 11 (13); case12 (14); case13 (15).

## 3 Case report

A 62-year-old male was presented to the Neurological Intensive Care Unit (NICU) of our hospital with recurrent GTCS accompanied by altered consciousness and urinary and bowel incontinence. On admission, brain magnetic resonance imaging (MRI) showed multiple bilateral lacunar infarcts and ischemic lesions (**Figure 2A**). Electroencephalogram (EEG) revealed mildly increased interictal epileptiform discharges in the bilateral frontal regions and global cerebral dysfunction (**Figure 3A**). CSF was colorless and clear with a slightly elevated opening pressure of 200 mmH_2_O (normal range: 80–180 mmH_2_O). CSF leukocyte count was normal at 1×10^6^/L (normal range: 0–8×10^6^/L), protein level was normal at 0.27 g/L (normal range: 0.15–0.45 g/L), glucose level was slightly elevated at 5.90 mmol/L (normal range: 2.3–4.1 mmol/L), and lactate level was slightly elevated at 7.3 mmol/L (normal range: 1.1–2.4 mmol/L). Given the acute encephalitic presentation and non-specific CSF abnormalities, infectious encephalitis, including viral encephalitis, was initially suspected, and the patient was commenced on empirical therapy consisting of acyclovir, ceftriaxone, and oral sodium valproate. After admission, he experienced intermittent seizures with progressive neurological deterioration and developed coma, requiring endotracheal intubation and mechanical ventilation. Cell-based assay (CBA) detected anti-KLHL11 antibodies (serum 1:100, CSF 1:30) (**Figure 4**). All other tested antibodies were negative, including: Autoimmune encephalitis antibodies: NMDAR, LGI1, CASPR2, AMPAR, GABABR, DPPX, IgLON5, GAD65, mGluR5, GlyR, and D2R; Demyelinating antibodies: AQP4, MOG, MBP, and GFAP; Paraneoplastic neuronal antibodies: Hu, Yo, Ri, CV2 (CRMP5), Amphiphysin, Ma1, Ma2, SOX1, Tr (DNER), Zic4, PKCγ, Recoverin, and Titin (MGT30); Ganglioside antibodies: SULFATIDE, GM1, GM2, GM3, GM4, GD1a, GD1b, GD2, GD3, GT1a, GT1b, and GQ1b. The patient received a diagnosis of anti-KLHL11 antibody encephalitis and was treated with intravenous immunoglobulin (IVIG) 0.4 g/kg/day for five days. High-dose corticosteroid pulse therapy was contraindicated due to his history of massive gastrointestinal bleeding and progressive decrease in hemoglobin levels. Following the above treatment, the patient’s consciousness gradually recovered. However, the patient had a history of multiple organ damage caused by benzidine poisoning at the age of 20, which resulted in hematuria and severe fatty liver disease. He also had long-standing underlying conditions including hypertension, diabetes mellitus, and cerebral infarction. Severe infection, sepsis, hypoalbuminemia, cardiac insufficiency, bilateral pleural effusion, deep vein thrombosis, and respiratory failure complicated the course, which remained critical and refractory. One month after admission, EEG showed scattered slow waves over bilateral frontotemporal regions during wakefulness, asynchronous between hemispheres and left-predominant. Scattered slow waves were also observed in the left occipital-posterotemporal and parietal regions (**Figure 3B**). Repeat MRI revealed acute cerebral infarction in the left periventricular region and adjacent corona radiata (**Figure 2B**). The patient also underwent additional tests including anti-phospholipase A2 receptor antibody, three rheumatology markers, five immune parameters, antinuclear antibody (ANA), antineutrophil cytoplasmic antibody (ANCA), immunofixation electrophoresis, human chorionic gonadotropin (hCG), and lactate dehydrogenase (LDH), all of which were unremarkable. It revealed no evidence of underlying malignancy by tumor marker testing, testicular ultrasound, contrast-enhanced CT of the whole abdomen, and PET-CT. The final diagnosis was anti-KLHL11 encephalitis without detectable malignancy. At the 2-month follow-up after discharge, the patient died secondary to central respiratory failure; the exact time of death was not provided.

**Figure 2:**
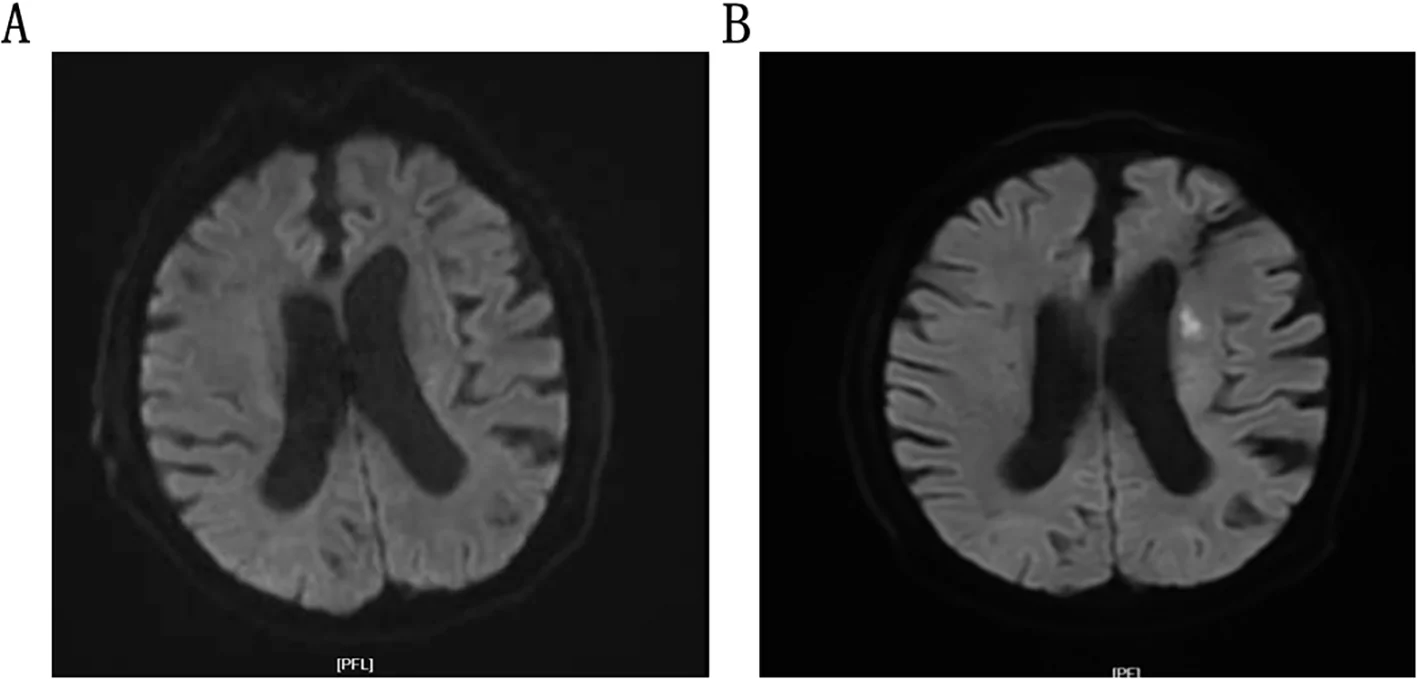
Brain MRI of the patient. **(A)** At admission. **(B)** One month after admission.

**Figure 3:**
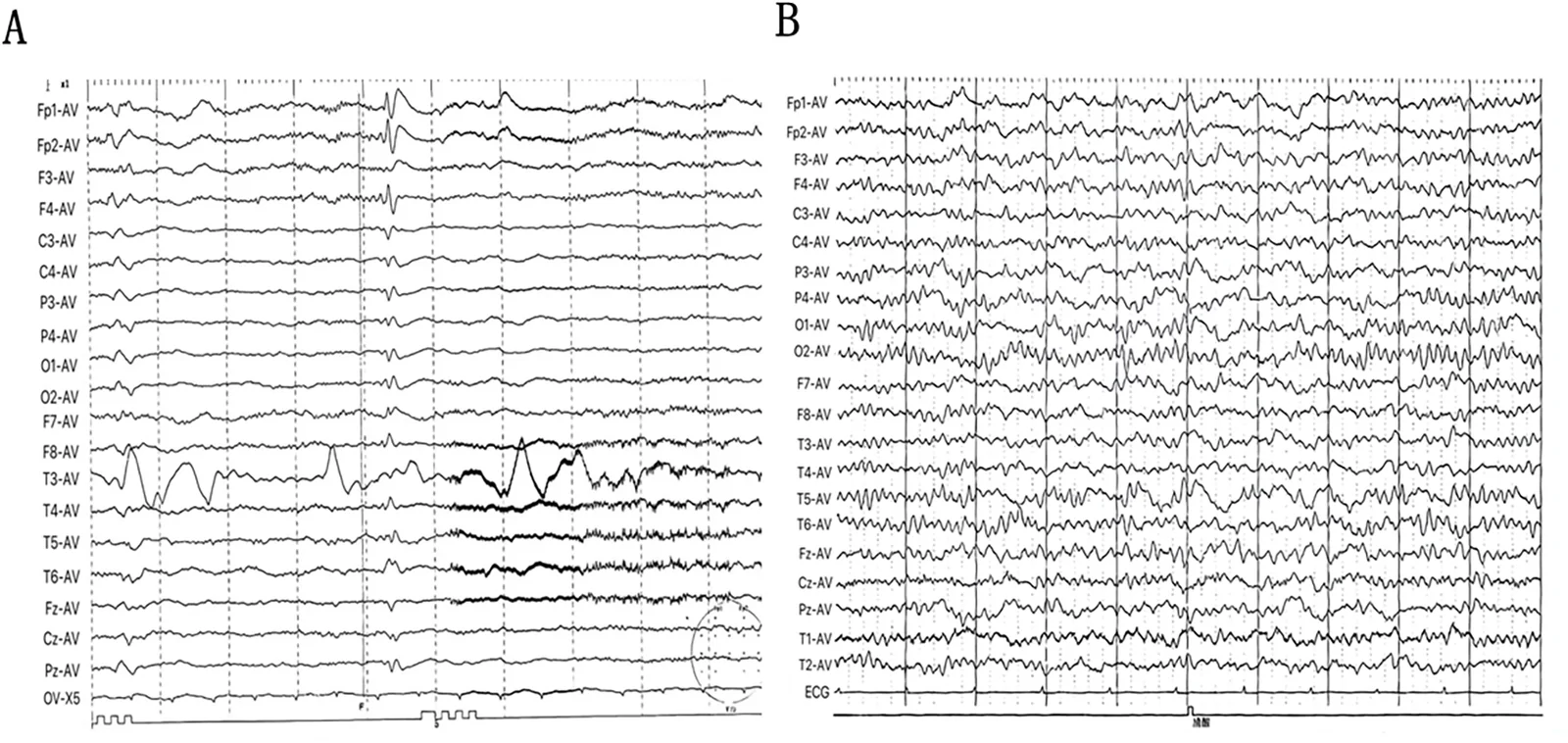
Electroencephalogram (EEG) of the patient. **(A)** At admission. **(B)** One month after admission.

**Figure 4:**
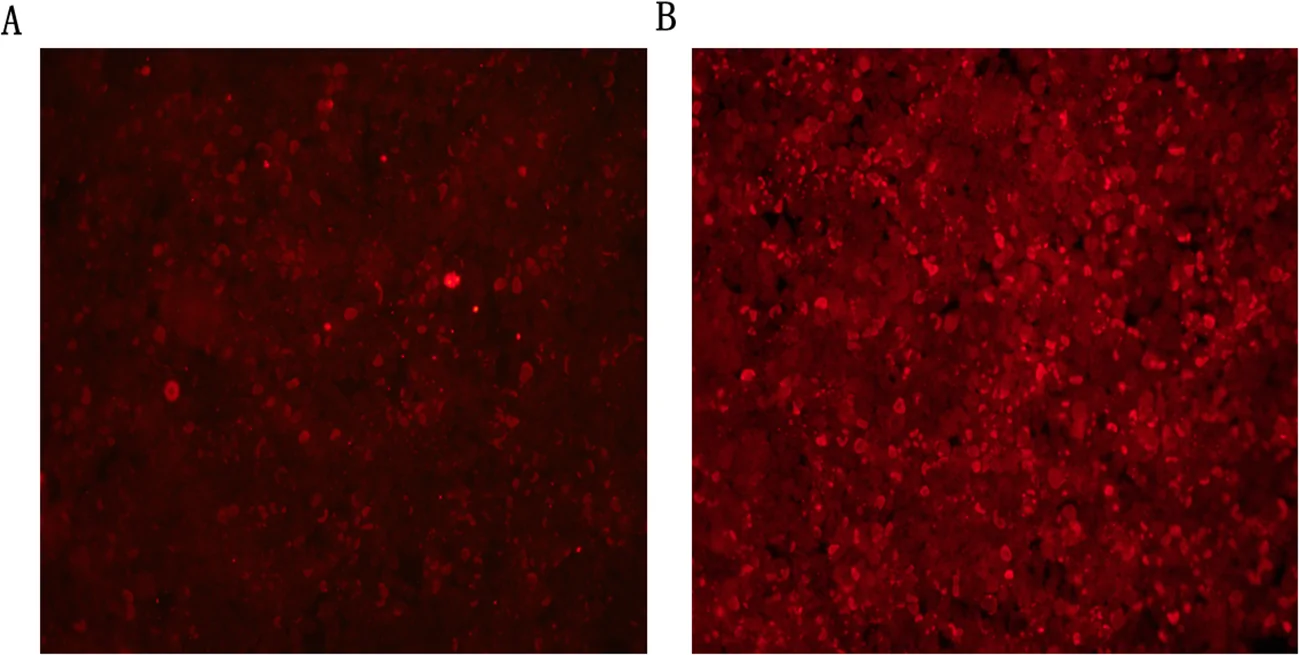
Anti-KLHL11 antibody titers in the index patient. **(A)** Anti-KLHL11 antibody titer in CSF (1:30); **(B)** Anti-KLHL11 antibody titer in serum (1:100).

## 4 Results of the literature search

### 4.1 Demographics and clinical characteristics

A total of 13 cases were included in this study, of which 76.9% (10/13) were male, and the mean age at onset was 54 years. The most common clinical manifestations were ataxia (69.2%) and dysarthria (61.5%), followed by gaze nystagmus (46.2%), vertigo, seizures, diplopia, and cognitive impairment (30.8% for each), and hearing loss (23.1%) (**Table 2**).

**Table 2:** Demographic and clinical data on all patients.

Characteristic	Value
Sex (male), n (%)	10 (76.9%)
Age (years), average	54
Clinical presentation, n (%)	
Ataxia	9 (69.2%)
Dysarthria	8 (61.5%)
Gaze/nystagmus	6 (46.2%)
Vertigo	4 (30.8%)
Epilepsy	4 (30.8%)
Diplopia	4 (30.8%)
Cognitive impairment	4 (30.8%)
Hypoacusis	3 (23.1%)
CSF	
Pressure	165 mmH2O
WBC, median	9x10^6^/L
Protein, median	0.5g/L
KLHL11, median	1:10
Serum	
KLHL11, median	1:100
MRI	
T2/FLAIR hyperintensity	7
Cerebellar/brainstem atrophy	4
Vascular lesions	1
Normal	3
Treatment, n (%)	
Glucocorticoids	8 (61.5%)
IVIG	7 (53.8%)
Plasma exchange	5 (38.5%)
Anti-CD20 mAbs	5 (38.5%)
Azathioprine	3 (23.1%)
Cyclophosphamide	2 (15.4%)
Efgartigimod	2 (15.4%)

### 4.2 Laboratory characteristics

CSF pressure was reported in 3 cases, ranging from 60 to 200 mmH_2_O, with a median of 165 mmH_2_O. CSF leukocyte count ranged from 1×10^6^/L to 46×10^6^/L, with a median of 9×10^6^/L, and the CSF protein level ranged from 0.27 g/L to 1.32 g/L, with a median of 0.50 g/L. KLHL11 antibodies were detected in both serum and CSF in 9 cases, and exclusively in serum in the remaining 4 cases. Serum KLHL11 antibody titers ranged from 1:30 to 1:160,000, with a median of 1:100, and CSF KLHL11 antibody titers ranged from 1:1 to 1:32, with a median of 1:10 (**Table 2**).

### 4.3 EEG

EEG was performed in 7 cases. Of these, 2 cases demonstrated normal EEG findings. Two exhibited unequivocal epileptiform discharges, with one affecting the bilateral frontal lobes and the other involving the bilateral temporal or sphenoidal regions. Diffuse background slowing was observed in 2 additional cases, while the remaining case manifested sleep-related central hypoventilation.

### 4.4 Neuroimaging findings

All 13 patients had comprehensive brain MRI examinations. Neuroimaging abnormalities were detected in 10 patients (76.9%, 10/13), while the remaining 3 patients (23.1%, 3/13) showed no detectable lesions. T2-weighted and FLAIR hyperintensities were identified in 7 patients (53.8%), which were categorized into three distinct patterns: 5 patients showed diffuse lesions involving the white matter and deep gray matter structures (case 6, 8, 9, 10, and 12); 1 patient had isolated limbic system involvement, characterized by T2 hyperintensities in the bilateral hippocampi and amygdalae (case 4); and 1 patient exhibited the characteristic salt-and-pepper sign, with punctate T2 hyperintensities scattered in the brainstem and cervical spinal cord (case 3). Cerebellar and/or brainstem atrophy was noted in 4 patients (30.8%), including 2 patients with isolated atrophy without signal abnormalities (case 2, 7) and 2 patients in whom atrophy coexisted with FLAIR hyperintensities (case 10, 12). Additionally, 1 patient (7.7%) had acute cerebral infarcts (**Table 2**).

### 4.5 Treatment and prognosis

Twelve of the 13 patients received immunotherapy. Treatments included IVIG (7/13), glucocorticoids (8/13, including methylprednisolone, prednisone, and prednisolone), plasma exchange (5/13), anti-CD20 antibodies (5/13), azathioprine (3/13), cyclophosphamide (2/13), and efgartigimod (2/13). Four patients received combination therapy with glucocorticoids and IVIG (**Table 2**). No patient attained complete remission after immunotherapy. Clinical improvement was observed in 6 patients, 3 remained stable, and 4 had clinical deterioration, among whom 2 died from central respiratory failure. Follow-up data were available for 10 patients, with a mean follow-up duration of 15 months. Among them, 5 showed clinical improvement, 2 remained stable, and 3 deteriorated, including 1 patient who died of central respiratory failure.

## 5 Discussion

Anti-KLHL11 encephalitis arises from cytotoxic T-cell-mediated neuronal injury. Dubey et al. (4) demonstrated that CD4+ helper T cells recognize the KLHL11 antigen via HLA class II molecules, triggering B-cell and CD8+ cytotoxic T-cell activation. Abundant CD8+ cytotoxic T cells infiltrate and attack KLHL11-expressing tumor cells and cerebral neurons, causing tumor regression and neurological injury. Vogrig et al. (16) suggested distinct inflammatory responses in testis (active anti-tumor) and brain (chronic exhaustion). In the brain, T cells gradually evolve from an initial active effector state to a chronic exhausted phenotype, causing neuronal loss and leading to irreversible brain atrophy and neurological dysfunction. The temporal relationship between T-cell-mediated neuronal injury and KLHL11 antibody levels may not be tightly coupled, which may explain why antibody titers do not consistently reflect clinical disease severity.

Anti-KLHL11 encephalitis is diagnosed by detecting antibodies in serum and/or CSF, preferably using tissue-based assay (TBA) and CBA (17). Consistent with this cellular immune-mediated mechanism, KLHL11 antibodies primarily serve as diagnostic biomarkers rather than direct mediators of neuronal injury, given that KLHL11 is an intracellular neuronal antigen. This mechanism parallels other paraneoplastic neurological syndromes that target intracellular neuronal antigens (e.g., anti-Hu, anti-Yo, anti-Ma2). Thus, KLHL11 antibody titers may not accurately reflect symptom severity or prognosis (18). Symptomatic improvement with efgartigimod (neonatal Fc receptor [FcRn] inhibitor) occurred without corresponding titer reduction (19); among two cases with initial serum titers of 1:30 and 1:100, respectively, the case with the lower titer had a rapidly progressive disease course and was refractory to multiple immunotherapies, and ultimately died (10). In contrast, in autoimmune encephalitides associated with antibodies targeting neuronal surface membrane antigens (e.g., anti-NMDAR encephalitis), antibodies directly drive disease pathogenesis, and persistent CSF antibody positivity is associated with relapse and long-term outcomes (20). However, the relationship between antibody titers and clinical severity remains a matter of ongoing debate. These observations suggest that the interpretation of antibody titers should take into account antigen specificity, localization, and underlying immunopathology. Vogrig et al. (16) proposed the MATCH score to identify KLHL11-IgG-positive patients. It consists of five components: M (male, 1 point), A (ataxia or other cerebellar signs, 1 point), T and C (testicular tumor, 2 points; or other tumor types, 1 point), H (hearing impairment, 1 point). A MATCH score threshold of ≥4 points is highly suspicious for KLHL11 encephalitis. Positive serum antibody testing yields 78% sensitivity and 99% specificity. Our patient scored 2 (male, 1; cerebellar signs, 1), below the diagnostic threshold. However, the patient had positive antibodies in both serum and CSF, which strongly suggests that for patients with high clinical suspicion but low scores, repeat tumor screening (including testicular ultrasound, PET-CT, and tumor markers) and KLHL11 antibody testing should still be performed to avoid missing the diagnosis of burned-out seminoma and to confirm anti-KLHL11 encephalitis without detectable malignancy.

Anti-KLHL11 encephalitis commonly co-occurs with malignancy. A study encompassing 133 reported cases showed that tumors were identified in 108 patients (81.2%). Testicular seminoma predominates, with ovarian teratoma, ovarian cancer, breast cancer, thymic germinoma, small cell lung cancer, lung adenocarcinoma, leukemia, Müllerian tumor, and esophageal cancer also reported (17, 21–23). In our case, no tumor was detected despite PET-CT, contrast-enhanced whole-abdomen CT, and testicular ultrasound. However, it is important to recognize that the absence of detectable malignancy may reflect either insufficient follow-up or the presence of occult malignancies, including burned-out (regressed) seminoma (24, 25). The classification of these cases as having no detectable malignancy was based on tumor screening during the available follow-up period rather than confirmed lifelong absence of malignancy. Some patients currently classified as having no detectable malignancy may actually harbor occult malignancy, particularly burned-out seminoma, resulting in potential misclassification and temporal heterogeneity across reported cases. Therefore, these classifications warrant careful interpretation, as future tumor detection remains possible. Long-term follow-up, periodic tumor screening, and testicular biopsy when indicated remain essential for these patients.

Studies have shown that anti-KLHL11 encephalitis typically presents with cerebellar syndrome and brainstem involvement; vestibulocochlear and limbic manifestations are less common (17). Common symptoms include ataxia, dysarthria, diplopia, nystagmus, vertigo, hearing loss, tinnitus, and seizures. Atypical features include psychiatric disorders, sleep disturbances, myelopathy, trigeminal neuralgia, and neck muscle atrophy resembling ALS (4, 16). In a summary of 13 cases without detectable malignancy, ataxia was the most frequent symptom (69.2%), consistent with tumor-positive KLHL11 encephalitis, suggesting a predilection for the cerebellar-brainstem circuit. The proportions of epilepsy and cognitive impairment (each 30.8%) were notably higher than those reported in patients with detectable malignancy, may suggest more prominent cortical and limbic system involvement in patients without detectable malignancy. Our patient presented initially with generalized tonic-clonic seizures, suggesting that KLHL11 encephalitis should be considered even in adult-onset unexplained epilepsy without cerebellar signs. Gaze palsy/nystagmus (46.2%) and diplopia (30.8%) reflect brainstem involvement, while vertigo (30.8%) and hearing loss (23.1%) suggest involvement of the vestibulocochlear pathway.

In addition to his atypical clinical presentation, this patient exhibited elevated CSF glucose and lactate levels, which are uncommon in autoimmune encephalitis. Such abnormalities may reflect hyperglycemia, sepsis, systemic metabolic disturbance, or tissue hypoperfusion in this patient. The presence of these non-specific CSF abnormalities, together with the patient’s acute encephalitic presentation, contributed to the initial suspicion of infectious encephalitis, including viral encephalitis. However, the etiology of these CSF abnormalities remains uncertain, and these atypical laboratory findings warrant further observation in future cases.

Anti-KLHL11 encephalitis lesions predominantly involve the cerebellum, brainstem and temporal lobe on MRI (10, 17). In this study, all 13 cases had brain MRI with dynamically evolving features across disease stages. Seven of 13 cases exhibited multifocal T2/FLAIR hyperintensities acutely, while four developed cerebellar and brainstem atrophy chronically. Two cases presented with both acute and chronic changes. These findings mirror the T cell-mediated neuronal injury mechanism of anti-KLHL11 encephalitis: early T cell infiltration causes inflammatory edema presenting as hyperintense signals, whereas sustained neuronal loss in the late phase leads to structural atrophy (4, 16). Our patient had atypical imaging manifestations. Admission MRI showed only old lacunar infarcts; repeat imaging at one month revealed new periventricular and corona radiata infarcts, possibly reflecting vascular compromise from systemic deterioration. Meanwhile, diffuse white matter and deep gray matter lesions (5/13) are rarely reported and merit attention. Seven cases underwent EEG, with an abnormal rate of 71.4%. Abnormalities included focal epileptiform discharges or background slowing. In addition, one patient had central hypoventilation, indicating the necessity of routine sleep respiratory monitoring for such cases.

No standardized treatment guidelines exist for anti-KLHL11 encephalitis. Current therapeutic strategies consist of underlying tumor management, immunotherapy, and symptomatic treatment (26). First-line immunotherapy includes intravenous methylprednisolone, intravenous immunoglobulin, and plasma exchange. Patients with inadequate first-line responses receive second-line therapies, including rituximab, cyclophosphamide, azathioprine, tacrolimus, ofatumumab, and efgartigimod. Broad-spectrum antiepileptic drugs control seizures. Management of anti-KLHL11 encephalitis remains challenging. Most patients fail first-line regimens, while novel targeted immunotherapies such as ofatumumab and efgartigimod achieve better outcomes in some cases (9, 19). Previous studies (4, 17) have demonstrated that few patients achieve full recovery. Nearly half suffer progressive neurological deterioration, with 28.8% of patients showing clinical improvement and 23.1% maintaining stable conditions. In our cohort, immunotherapy improved symptoms in nearly half during follow-up. Our patient presented comatose but recovered consciousness after intravenous immunoglobulin and valproate. However, the patient subsequently developed multiple life-threatening complications, including severe infection, sepsis, hypoalbuminemia, cardiac dysfunction, bilateral pleural effusions, deep vein thrombosis, and respiratory failure. Acute infarcts adjacent to the lateral ventricles and corona radiata developed one month after admission, and the patient died of central respiratory failure. We speculate that the cause of death in this patient likely reflects the convergence of multiple pathological processes rather than a single underlying mechanism. It remains uncertain whether the acute cerebral infarction identified in this patient was related to a vascular complication of anti-KLHL11 encephalitis. Several contributing factors should be considered, including sepsis-associated systemic inflammation and hypoperfusion (27), as well as progression of pre-existing cerebrovascular disease. Meanwhile, severe infection progressing to sepsis, cardiac insufficiency, and bilateral pleural effusion further exacerbated respiratory impairment and systemic circulatory dysfunction, collectively contributing to end-stage respiratory failure. In addition, the patient’s preexisting multiple organ damage secondary to benzidine poisoning may have reduced his tolerance to severe infection and neurological injury. This course suggests that the interaction between neurological injury and severe systemic complications may still determine the final outcome in patients with anti-KLHL11 encephalitis burdened by severe comorbidities and fragile systemic reserve, despite transient neurological improvement from immunotherapy.

## 6 Conclusion

Anti-KLHL11 encephalitis without detectable malignancy is a rare disorder that is difficult to distinguish from tumor-positive cases based solely on clinical manifestations and neuroimaging findings. Extensive white matter and deep gray matter involvement, together with prominent seizures, may be more prevalent in patients without detectable malignancy, although these observations require validation in larger comparative cohorts. Comprehensive tumor screening and prolonged follow-up remain essential even when initial tumor workup is negative. Although immunotherapy may improve neurological function, management of complications critical to the prognosis and should be incorporated early.

## Data Availability

The original contributions presented in the study are included in the article/supplementary material. Further inquiries can be directed to the corresponding author.
